# The impact of osteoarthritis on early exit from work: results from a population-based study

**DOI:** 10.1186/s12889-018-5381-1

**Published:** 2018-04-11

**Authors:** Pedro A. Laires, Helena Canhão, Ana M. Rodrigues, Mónica Eusébio, Miguel Gouveia, Jaime C. Branco

**Affiliations:** 1 Faculdade de Medicina da Universidade de Lisboa, Lisbon Academic Medical Center, Lisbon, Portugal; 2 Centro de Investigação em Saúde Pública, Escola Nacional de Saúde Pública, Lisbon, Portugal; 3 EpiReumaPt Study Group, Sociedade Portuguesa de Reumatologia, Lisbon, Portugal; 4 Chronic Diseases Research Center (CEDOC), NOVA Medical School, Universidade Nova de Lisboa (NMS/UNL), Lisbon, Portugal; 5 Catolica Lisbon School of Business and Economics, Lisbon, Portugal; 6 Rheumatology Department, Hospital Egas Moniz, Centro Hospitalar Lisboa Ocidental (CHLO- E.P.E.), Lisbon, Portugal

**Keywords:** Exit from work, Early Retirement, Work disability, Indirect costs, Cost of illness, Economic impact, Health Survey, Osteoarthritis

## Abstract

**Background:**

Osteoarthritis (OA) is a leading cause of pain and disability, which may be a source of productivity losses. The objectives of this study were to describe the impact of OA, namely through pain and physical disability, on early exit from work and to calculate its economic burden.

**Methods:**

We analysed data from the national, cross-sectional, population-based EpiReumaPt study (Sep2011–Dec2013) in which 10,661 individuals were randomly surveyed in order to capture all cases of rheumatic diseases. We used all participants aged 50–64, near the official retirement age, who were clinically validated by experienced rheumatologists (*n* = 1286), including OA cases. A national database was used to calculate productivity values by gender, age and region, using the human capital approach. The impact of OA on the likelihood of early exit from work and the population attributable fractions used to calculate due economic burden (indirect costs) were obtained at the individual level by logistic regression. All results were based on weighted data.

**Results:**

Almost one third of the Portuguese population aged 50–64 had OA (29.7%; men: 16.2% and women: 43.5%) and more than half were out of paid work (51.8%). Only knee OA is associated with early exit from work (OR: 2.25; 95%CI: 1.42–3.59; *p* = 0.001), whereas other OA locations did not reach any statistical difference. Furthermore, we observed an association between self-reported longstanding musculoskeletal pain (OR: 1.55; 95%CI: 1.07–2.23; *p* = 0.02) and pain interference (OR: 1.35; 95%CI: 1.13–1.62; *p* = 0.001) with early exit from work. We also detected a clear relationship between levels of disability, measured by the Health Assessment Questionnaire (HAQ), and the probability of work withdrawal. The estimated annual cost of early exit from work attributable to OA was €656 million (€384 per capita; €1294 per OA patient and €2095 per OA patient out-of-work).

**Conclusions:**

In this study, we observed an association between OA and early exit from work, largely dependent on pain and disability. This relationship translates into a meaningful economic burden amounting to approximately 0.4% of the national Gross Domestic Product (GDP). The high prevalence and the impact of this disabling chronic disease highlight the need to prioritize policies targeting early exit from work in OA.

## Background

Osteoarthritis (OA) is a degenerative joint disorder and the most common form of arthritis in adults [1]. It is characterized by pain and functional impairment, which may lead to disability, including work restriction [2–4].

In many western countries the population is ageing due to increasing longevity and falling birth rates [5]. Portugal, for instance, is amongst the oldest countries in the world, and has one of the highest old-age dependency ratios, further aggravated by the fact that currently unemployment and overall premature work withdrawal are still high [6].

Numerous factors, including health-related problems, may contribute to the high rate of exit from the workforce that persists at a global level [7–11]. In fact, several studies have already shown that ill-health is a risk factor for early exit from work, including retirement and unemployment [12, 13, 16]. A deeper understanding of these factors is crucial to support policies for increasing productivity and postponing exit from work.

The growing prevalence of chronic disabling diseases, such as OA, jeopardizes any effort to prolong the working life of the labour force of modern societies. In spite of this, research about the effect of OA on work participation and its economic burden is still lacking [14, 15].

Thus, the objectives of this study were to describe the impact of OA, namely through pain and physical disability, on early exit from work and to calculate its economic burden.

## Methods

### Sample

This study uses the first national, cross-sectional, population-based study on rheumatic diseases in Portugal – the EpiReumaPt study [16]. The methodology of EpiReumaPt has been detailed elsewhere [17]. Briefly, EpiReumaPt (September 2011–December 2013) randomly selected a representative sample of 10,661 adult subjects who self-reported several data following a questionnaire applied by trained staff. Rheumatologists then performed a standardized physical examination and appropriate laboratory/imaging evaluation on those participants with rheumatic complaints/symptoms.

For the purposes of this analysis we used data from EpiReumaPt participants aged between 50 and 64 years old, near the official retirement age, who were clinically evaluated by the rheumatologists (*n* = 1286).

### Measures

We considered the presence of OA through clinical confirmation initially done by a rheumatologist and then validated by a team of three experienced rheumatologists, in accordance with the American College of Rheumatology (ACR) classification criteria, of at least one type of OA: knee OA [18], hip OA [19], and hand OA [20]. All those without any of these OA types were considered to be non-OA.

The following self-reported measures were used: major chronic diseases (rheumatic diseases, diabetes, cardiovascular diseases including risk factors such as hypertension and high cholesterol, allergy, pulmonary disease, gastrointestinal disease, neurological disease, mental disease, and cancer); comorbidity score (built as the sum of all aforementioned chronic illnesses); body mass index (BMI, derived from self-reported height and weight and categorized as underweight: < 18.5m^2^/kg, normal: 18.5-25 m^2^/kg, overweight: 25-30 m^2^/kg and obese: ≥30 m^2^/kg); quality of life (using the final 0–100 score from the SF-36 Health Survey [21, 22] and its eight items separately, including the bodily pain; and the European Quality of Life questionnaire with five dimensions and three levels validated for Portugal, EQ-5D index [23, 24]); longstanding musculoskeletal pain (≥3 months); pain interference with function (i.e. pain affecting labour and domestic activities based on the SF-36 question: *During the past 4 weeks, how much did pain interfere with your normal work, including both work outside the home and housework?*); functional capacity (0–3 score from the Health Assessment Questionnaire, HAQ [25]); marital status; household monthly income; and educational level (classified into three major levels according to the highest degree completed: primary school or less, basic education between primary and secondary levels, and secondary education or more, including university degrees). Since the underlying etiological model of this research, which connects OA with early exit from work, comprises pain and disability as important intermediate steps, we analysed them in further detail by using the abovementioned instruments.

Regarding the case definition for the main outcome, individuals were asked directly about their employment status and all those who did not report any kind of paid work (part- or full-time), including students, housekeepers or anyone without a regular salary; as well as those in official early retirement or disability pensions, were included in the early exit from paid employment group. All those reporting any form of regularly paid work were considered employed. This definition was used elsewhere in earlier research [1, 26].

A broader definition of early withdrawal from work allows us to take into account the existence of different pathways to early retirement. However, we also considered it relevant to separately analyse more specific types of exit from work, including official early retirement and unemployment.

### Economic burden (INDIRECT COSTS)

In order to calculate the productivity loss (indirect costs) of early exit from work attributable to OA in Portugal’s mainland we adopted the society’s perspective, which considers the aggregate value of indirect costs for all parts in the Society (including patients, employers, and the government), and used the human capital approach. This method estimates productivity by valuing healthy time lost due to OA using market wage rates, which can be viewed as the loss of an investment in a person’s human capital [27]. Early exit from paid employment associated with OA was assessed through logistic regression models. A good measure of the impact of OA in the early exit from work may be the population attributable fractions (PAF), which take into account both the strength of the association between OA and early exit from work, as measured in the logistic regression models, and the prevalence of OA in the population surveyed. PAF were calculated as the resulting proportional change in the probability of premature work withdrawal following a counterfactual exercise in which the presence of OA was artificially eliminated from the sample. This recalculated probability of exit from work was then used to estimate the indirect costs attributable to OA by multiplying each observation’s probability change with the corresponding unit value of production. The unit values of production were assessed by obtaining the market wage rates from national public sources [28]. These figures needed to be grossed up by social security contributions. This approach yielded an annual average value of €24,891 for men and €16,079 for women, for ages between 50 and 64. All unit values of production were stratified by age, gender, and geographic region (Appendix 1).

### Statistical analysis

Descriptive analysis was mainly performed by comparing early withdrawals from work (including early retirees) versus the employed participants of the sample analysed. Prevalence of early exit from work and other characteristics were computed as weighted data, in order to take into account the stratified sampling design of the survey. Missing values were not replaced and all statistics were calculated based on non-missing values. To explore the association between OA and early exit from work we built multivariable logistic regression models by means of a manual stepwise technique (backward elimination) using the following potential confounders: age, gender, region, marital status, education, household income, BMI, and chronic diseases. Pain and disability were not included in the multivariable models since they are considered intermediate factors of OA within the etiologic model adopted [29, 30]. Their association with the outcome was instead analysed separately adjusted for age, gender, and region. All statistical analyses were carried out using Stata IC V.12 (StataCorp, 2011. Stata Statistical Software: Release 12. College Station, Texas, USA: StataCorp LP).

## Results

Amongst the survey respondents, more than half of the population aged between 50 and 64 years were out of paid work (51.8%. Table 1) and had an OA prevalence of about 30% (29.7%; men: 16.2% and women: 43.5%. Knee OA: 18.6%; hand: 12.6%; hip: 3.6%). Lower education and monthly household income were more frequent in the premature withdrawals, whilst the marital status single was more likely found in the employed. The out-of-work group had a poorer health status, with higher comorbidity scores, lower quality of life, and lower functional capacity. Higher prevalence of diabetes and neurological disorders were also found in this group. In comparison to the paid employment group, OA prevalence was significantly higher in all other groups (Table 1).

**Table 1:** General description of the sample by employment status: EpiReumaPt ages 50–64 (*n* = 1286)

	UNEMPLOYMENT	EARLY RETIREMENT	EXIT FROM WORK	PAID EMPLOYMENT
Employment Status	12.2%	30.2%	51.8%	48.2%
Age (years)	55.2 (*p =* 0.192)^*^	59.9 (*p <* 0.001)^*^	58.2 (*p <* 0.001)^*^	54.6
Gender (female)	55.3% (*p =* 0.187)	44.2% (*p =* 0.757)	52.2% (*p =* 0.305)	46.3%
Marital Status				
Single	11.7%	3.4%	5.7%	14.9%
Married/Consensual union	70.6%	77.3%	76.2%	64.5%
Divorced	14.3%	10.4%	10.3%	15.3%
Widowed	3.4% (*p =* 0.673)	8.9% (*p =* 0.002)	7.8% (*p =* 0.007)	5.2%
Body Mass Index				
Underweight (< 18.5m^2^/kg)	0%	0.5%	0.5%	0.4%
Normal (18.5-25 m^2^/kg)	28.5%	25.7%	27.0%	30.5%
Overweight (25-30 m^2^/kg)	44.6%	50.3%	46.3%	46.7%
Obese (≥30m^2^/kg)	27.7% (*p =* 0.788)	23.5% (*p =* 0.801)	26.2% (*p =* 0.762)	22.4%
Clinically Confirmed OA	42.8% (*p <* 0.001)	32.1% (*p =* 0.08)	35.4% (*p =* 0.003)	23.5%
Knee OA	29.0% (*p <* 0.001)	22.0% (*p =* 0.005)	24.2% (*p <* 0.001)	12.6%
Hand OA	14.3% (*p =* 0.413)	12.3% (*p =* 0.797)	13.6% (*p =* 0.405)	11.6%
Hip OA	3.1% (*p =* 0.795)	4.2% (*p =* 0.809)	3.6% (*p =* 0.998)	3.6%
Chronic Diseases (self-reported)				
Cardiovascular	60.6% (*p =* 0.740)	62.8% (*p =* 0.991)	63.7% (*p =* 0.874)	62.8%
Diabetes	10.1% (*p =* 0.180)	17.5% (*p =* 0.003)	15.9% (*p <* 0.001)	6.4%
Pulmonary	11.5% (*p =* 0.147)	7.5% (*p =* 0.407)	9.9% (*p =* 0.110)	5.0%
Allergy	22.4% (*p =* 0.411)	20.5% (*p =* 0.242)	22.4% (*p =* 0.356)	27.5%
Gastrointestinal	22.6% (*p =* 0.460)	26.6% (*p =* 0.114)	25.5% (*p =* 0.102)	19.1%
Neoplasic	5.9% (*p =* 0.458)	6.5% (*p =* 0.282)	5.7% (*p =* 0.338)	3.9%
Mental	15.8% (*p =* 0.493)	13.6% (*p =* 0.942)	17.2% (*p =* 0.130)	13.4%
Neurologic	1.6% (*p =* 0.644)	6.7% (*p =* 0.005)	4.8% (*p =* 0.004)	1.2%
Comorbidity Score	1.87 (*p =* 0.471)	2.03 (*p =* 0.10)	2.04 (*p =* 0.03)	1.74
Longstanding Musculoskeletal Pain	47.0% (*p =* 0.049)	39.7% (*p =* 0.399)	43.9% (*p =* 0.073)	34.8%
Functional Capacity - HAQ	0.48 (*p =* 0.012)	0.43 (*p =* 0.033)	0.47 (*p =* 0.002)	0.28
Quality of Life – EQ-5D	0.72 (*p <* 0.001)	0.79 (*p =* 0.152)	0.75 (*p =* 0.002)	0.83
Educational Level				
Primary or less	55.0%	64.0%	63.4%	37.3%
Medium	23.3%	17.6%	19.6%	28.5%
High	21.7% (*p =* 0.024)	18.4% (*p <* 0.001)	17.0% (*p <* 0.001)	34.2%
Monthly Household Income				
≤ €500	49.2%	22.3%	29.8%	12.1%
€501–€1000	36.3%	37.6%	40.7%	38.6%
€1001–€2000	8.5%	28.7%	20.7%	33.1%
> €2000	6.0% (*p <* 0.001)	11.4% (*p* = 0.243)	8.8% (*p =* 0.001)	16.2%

*All results were computed as weighted proportions to keep in account the sampling design of the survey*

*all p values versus paid employment. OA, Osteoarthritis; HAQ, Health Assessment Questionnaire; EQ-5D, European Quality of Life index

The participants with OA had more comorbidities (Comorbidity score: 2.3 vs 1.7; *p* < 0.001. Table 2), in particular diabetes, gastrointestinal and mental disorders, and were more likely to self-report other main chronic diseases (age, gender, and region adjusted OR: 1.70; CI: 1.07–2.69; *p* = 0.023). OA participants had poorer quality of life measured by all SF-36 items, especially the bodily pain index (54.9 vs. 72.6, *p* < 0.001), and by the EQ-5D score (0.68 vs. 0.83; *p* < 0.001). OA patients more frequently reported longstanding musculoskeletal pain than non-OA (52.5% vs. 34.1%; p < 0.001). The OA population also scored worse in functional capacity measured by the HAQ scale (0.64 vs. 0.28; *p* < 0.001). This is especially true for the knee OA location (mean score: 0.61, 95%CI: 0.53–0.69). Within the OA group, 61.8% were not working versus 47.6% for those without OA (*p* = 0.004). Most were females (71.0% versus 41.9% for those non-OA in the out-of-work group; *p* < 0.001). A non-significant difference was observed when analysing early retirement specifically (32.6% vs. 29.1%, respectively; *p* = 0.437. Table 2). Unemployment is a major early route of work loss in the OA population (17.7% vs. 9.9% for non-OA; *p* = 0.002). The vast majority of registered unemployment lasted more than 12 months (95.8%).

**Table 2:** Description of the sample by OA diagnosis, including pain, functional status and quality of life results (SF-36 and EQ-5D): EpiReumaPt ages 50–64 (*n* = 1286)

	OA (*n* = 382)	NON-OA (*n* = 904)
Age (years)	57.5	56.0 (*p <* 0.001)^*^
Gender (female)	72.3%	39.6% (*p* < 0.001)
Employment Status		
Unemployment	17.7%	9.9% (*p* = 0.002)
Early Retirement	32.6%	29.1% (*p* = 0.437)
Exit from Work	61.8%	47.6% (*p* = 0.004)
Educational Level		
Primary or less	57.9%	47.9%
Medium	20.2%	25.4%
High	21.8%	26.7% (*p* = 0.148)
Body Mass Index		
Underweight (< 18.5m^2^/kg)	0.5%	0.4%
Normal (18.5-25 m^2^/kg)	26.9%	29.5%
Overweight (25-30 m^2^/kg)	42.3%	48.3%
Obese (≥30m^2^/kg)	30.3%	21.8% (*p =* 0.207)
Chronic Diseases (self-reported)		
Cardiovascular	68.9%	61.0% (*p =* 0.108)
Diabetes	15.5%	9.5% (*p =* 0.027)
Pulmonary	7.7%	7.5% (*p =* 0.962)
Allergy	29.7%	22.8% (*p =* 0.158)
Gastrointestinal	32.0%	18.3% (*p* < 0.001)
Neoplasic	4.0%	5.2% (*p =* 0.454)
Mental	19.3%	13.8% (*p =* 0.029)
Neurologic	3.5%	2.9% (*p =* 0.602)
Comorbidity Score	2.3	1.7 (*p* < 0.001)
Longstanding Musculoskeletal Pain	52.5%	34.1% (*p* < 0.001)
SCORE HAQ *(0–3 from best to worst)*	0.64	0.28 (*p* < 0.001)
SCORE EQ-5D *(0–100 from worst to best imaginable health state)*	0.68	0.83 (*p* < 0.001)
SF-36 physical function *(0–100 from worst to best)*	65.2	80.8 (*p* < 0.001)
SF-36 role limitations because of physical health problems	59.8	77.3 (*p* < 0.001)
SF-36 bodily pain	54.9	72.6 (*p* < 0.001)
SF-36 social functioning	80.3	87.8 (*p* < 0.001)
SF-36 general mental health	58.8	69.7 (*p* < 0.001)
SF-36 role limitations because of emotional problems	71.2	85.3 (*p* < 0.001)
SF-36 vitality	51.4	61.9 (*p* < 0.001)
SF-36 general health perceptions	47.8	56.6 (*p* < 0.001)

*All results were computed as weighted proportions to keep in account the sampling design of the survey*

*All p values: OA versus non-OA. OA, Osteoarthritis; HAQ, Health Assessment Questionnaire; EQ-5D, European Quality of Life index; SF-36, Short-Form Health Survey

**Fig. 1:**
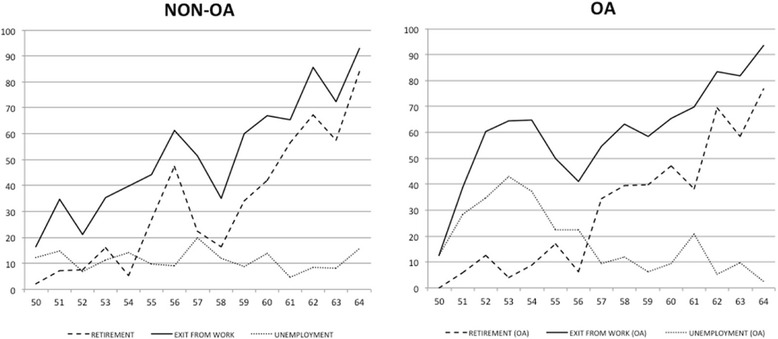
Prevalence of Early Exit from Work, Early Retirement and Unemployment according to ages 50–64 (*n* = 1286). All results were computed as weighted proportions to keep in account the sampling design of the survey. OA, Osteoarthritis

Figure 1 shows how unemployment rates were high amongst the youngest of the OA population (i.e. 50–56) whilst being surpassed by official early retirement after approximately the age of 56. A distinct dynamic is observed in the non-OA population, in which unemployment rates are in line with early retirement below age 55.

### Association between OA and early exit from work

OA is associated with early exit from work (OR: 1.85; 95%CI: 1.27–2.69; *p* = 0.001), but not with official early retirement (OR: 1.43; 95%CI: 0.96–2.12; *p* = 0.08). Knee location of OA is strongly associated with early exit from work (OR: 2.25; 95%CI: 1.42–3.59; *p* = 0.001. Appendix 2), while no significant association was observed for hand OA (OR: 1.17; 95%CI: 0.76–1.80; *p* = 0.477) or hip OA (OR: 1.04; 95%CI: 0.36–2.99; *p* = 0.938). As mentioned above, unemployment seems to be a major channel of exit from work for patients with OA (OR: 1.97; 95%CI: 1.27–3.06; *p* = 0.002), especially for knee OA (OR: 2.68; 95%CI: 1.58–4.53; *p* < 0.001. Table **2**), and for the youngest (50–57) from the sample (OR: 3.47; 95%CI: 1.88–6.41; *p* < 0.001).

### Pain and disability

**Fig. 2:**
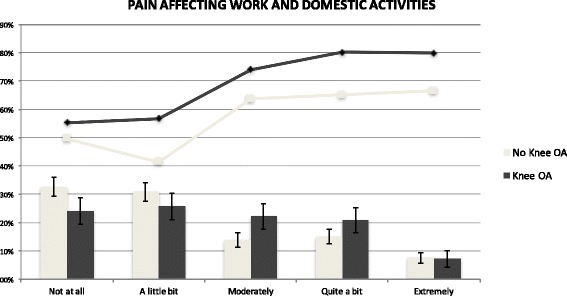
Pain Affecting Work and Domestic Activities & Early Exit from Work (n = 1286). *All results were computed as weighted proportions to keep in account the sampling design of the survey. Lines indicate Early Exit from Work (all p-values of Knee OA versus No Knee OA groups are non-significant)*. Bars indicate Pain Interference distribution by intensity levels (*p* = 0.02) with corresponding 95% confidence intervals (vertical lines). OA, Osteoarthritis

Pain plays a key role in the risk of workforce withdrawal. A strong association was seen between pain interference and premature work loss, especially within the knee OA population (OR: 1.52; 95%CI: 1.16–1.99; *p* = 0.002). Those who scored worse in pain interference were more often out-of-work (Fig. 2). In fact, not only was the OA population more likely to score worse (*p* = 0.02) in this parameter, but also the aforementioned association between knee OA and exit from work becomes non-significant if only the subset of population with low pain interference is analysed (i.e. “not at all” “to a little bit” pain interference. OR: 1.52; 95%CI: 0.95–2.44; *p* = 0.08).

Furthermore, knee OA patients with the highest levels of disability measured by HAQ (scores ≥2), are at the greatest risk of early exit from work (80.7% vs. 67.4% for all knee OA population and 51.2% for those with HAQ scores ≥2 but without knee OA). We estimated an almost linear relationship between levels of disability and the probability of early exit from paid employment, with its y-intercept increasing with the presence of knee OA (Appendix 3). Similarly to what was observed with pain, the association between knee OA and exit from work becomes non-significant if only the subset of population with less disability is analysed (i.e. HAQ scores below the average of the population [0.38]. OR: 0.74; CI: 0.28–1.99; *p* = 0.552).

### Economic burden

**Fig. 3:**
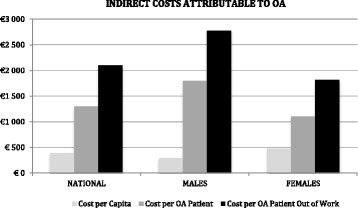
Indirect costs per capita in the Portuguese population aged 50–64, per OA patient and per OA patient out of work (*n* = 1286). OA, Osteoarthritis

The estimated annual indirect cost due to premature exit from work attributable to OA was €656 million (€384 per capita; €1294 per OA patient and €2095 per OA patient out-of-work). Females contributed with 61.6% of these costs (€404 million), but mean per capita indirect costs were greater for OA males (cost per OA male patient: €1795 vs. €1102 for females; cost per OA male patient out-of-work: €2776 vs. €1817 for females Fig. 3).

## Discussion

In this study, using the first nationwide epidemiological population-based survey that evaluated rheumatic diseases in Portugal, we found an association between clinically confirmed OA and early exit from paid employment. However, this relationship was only found for the knee OA (not for hip or hand OA). We also estimated that OA might have led to annual indirect costs amounting to approximately 0.4% of the 2013 national Gross Domestic Product (GDP). Women contributed with 61.6% of this productivity loss, whilst OA men have higher mean *per capita* indirect costs given the higher average unit values of production associated with male gender [31].

As expected [1], OA patients are mostly ageing females when compared with the non-OA population. They have lower levels of education, lower household income, poorer self-reported quality of life, and a higher number of comorbidities. These characteristics may themselves influence labour force participation. In fact, we observed an association between premature work loss and lower levels of education, marital status (married or widowed), neurological diseases, and lower household income. Nevertheless, the association we found between clinically confirmed OA and premature work withdrawal is robust and independent of other influencing factors, which is consistent with previously published data [32–34].

OA generates disabling pain, which might push patients to leave work prematurely [14]. We captured pain through alternative measures (i.e. pain interference, bodily pain item from the SF-36 questionnaire, and self-reported longstanding musculoskeletal pain) and, as expected, OA patients consistently report more pain than others without the condition. In addition, we observed a relation between pain and withdrawal from employment. This is consistent with the literature [32, 35, 36]. For instance, Dibonaventura et al., using data from the US National Health and Wellness Survey, have shown that individuals with OA pain were less likely to be employed relative to workers without OA pain [35]. We also detected that OA patients with higher levels of disability, measured by high scores of HAQ, were at the highest risk of early exit from work. This also aligns with earlier research [37, 38] and with the etiological model adopted, which assumes that OA generates pain, impairment, and disability that may ultimately lead to work withdrawal. Thus, we confirmed in our research and study population that pain and disability are key factors for job loss driven by OA.

Interestingly, we did not find a statistical association between OA and official early retirement (regardless of anatomic location). OA seems instead to drive the labour force out of paid employment mainly through unemployment. This is particularly evident in the earlier ages. Thereafter unemployment is overtaken by early retirement when patients with OA get closer to the official retirement age. We did not find the same interplay between age-specific frequencies of retirement and unemployment in the non-OA population. Thus, unemployment appears to be the predominant first step to irreversible out-of-work state in OA patients, although likely not being fully converted into official early retirement, given the lack of association between early retirement and OA (in Portugal, under some circumstances, unemployment benefits may be converted into the old-age pension before reaching the statutory retirement age).

It would be valuable to further explore if this occurs because labour market policies are restricting early retirement in this sort of patients (e.g. formal rejection of early retirement applications to Social Security from employees with OA) or if other reasons are taking the lead instead. This finding highlights the need to target research and integration-oriented policies toward unemployment generated by OA. More knowledge in this area may produce employment gains since premature retirement restriction policies by themselves are insufficient to mitigate the job losses, as alternative routes may take place as seen herein in the OA case. Although unemployment benefits may be time limited, from the societal perspective these pathways of early exit from work embody the same economic burden (i.e. identical productivity losses), and therefore strategies that simply push individuals from one route to another are truly not socially efficient.

In this nationwide study, we estimated a substantial economic burden following early exit from work attributable to OA, equivalent to an indirect annual cost of €656 million euros (€2061 per patient). Earlier research in other countries has measured different yearly indirect costs of OA per patient [39–46]. Comparing results of cost-of-illness studies is hampered by discrepancies across study designs, case definition, methodological choices, wage levels, and sources of data used [47]. Additionally, these cost results vary with time and geography. However, there are some common take-away messages: First, the economic burden of osteoarthritis is considerable and closely related to its high prevalence. Second, indirect costs are likely to surpass other per patient costs (e.g. drug and other medical costs) [42, 48, 49]. Third, these costs are also likely to exceed those from other chronic disabling conditions. For example, Schofield et al. measured annual losses of arthritis through early retirement (approximately 0.7% of GDP) [50] superior to other pathologies, such as cardiovascular disease [51], diabetes [52], and mental disorders [53].

This study has several limitations that must be taken into account when interpreting its results. First, it may be limited by the cross-sectional design, which does not allow for an evaluation of the temporal relationship between onset of OA and time of exit from paid employment, which would help in establishing a cause–effect relationship. Lack of available national longitudinal data made this limitation impossible to overcome. Nevertheless, onset of OA is likely to start before premature exit from paid employment and reverse causality is unlikely (i.e. early exit from work as a risk factor to OA onset). Second, wages were estimated through official statistics based on gender, region, and age. Necessarily, this methodology is a rough estimate of the individual unit values of production. In particular, OA patients are likely associated with lower incomes. Still, given the information available, it is the best feasible method. Third, due to the cross-sectional design, our results are right-censored, leading to likely underestimation of the results (i.e. further exit from work is more probable to occur than return to work within the sample until all participants reach the official retirement age). Finally, not all potential hidden confounding factors could be addressed. However, we do not consider our analysis to be overly influenced by this limitation given the richness of the EpiReumaPt dataset and the large number of control variables thus afforded.

This study has several strengths as well. It is based on the largest population-based study about rheumatic diseases ever performed in Portugal (i.e. high external validity). It uses confirmed diagnosis of OA done by rheumatologists (i.e. very strict and controlled case definition). To our knowledge, this must be amongst the few studies focusing on indirect costs of OA based on such a representative sample specifically dedicated to rheumatic and musculoskeletal conditions, a sample that is likely to accurately reflect this particular type of economic impact on society. It will certainly facilitate future research on the cost-effectiveness of interventions targeting reduced work ability due to OA.

Unquestionably, this issue deserves further attention. Recently, access to early retirement was restricted in some European countries that are still lacking in integration policies such as vocational rehabilitation programmes [54]. Since OA is highly disabling and prevalent, especially at ages near the statutory retirement age, it might well be an ideal area in which public policies might strive for a better balance between restricting early retirement and integration policies. Moreover, OA is a musculoskeletal disease for which the etiological model (from the onset of symptoms/pain until work withdrawal) may be further understood and subsequently disrupted by effective health interventions.

## Conclusions

This population-based study unveils the impact of the association between OA and early exit from paid employment. It describes the high economic burden underlying this association. The findings justify giving more attention to OA when discussing policies facing the ageing of higher income countries. The depreciation in the stock of human capital due to OA is already extensive, and given the demographic and epidemiological trends, it may even worsen if nothing is done. This research should provide important evidence for decision makers to prioritize investments in health and policies targeting patients with OA.

## Figures and Tables

**Fig. 4:**
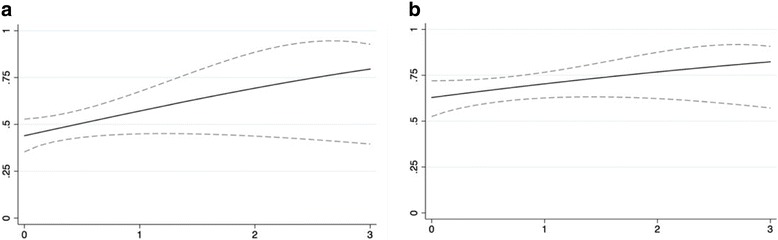
Relationship between disability levels and probability of early exit from work (by presence of knee OA). **a** Without knee OA; **b** With knee OA. OA, osteoarthritis; HAQ, Health Assessment Questionnaire. Median bands are depicted in solid lines, while the confidence interval consists of the space between the two dashed lines

## Appendices

### Appendix 1

**Table 3:** Summary of average unit values of production by gender, age and geographic region (2013)

Age	National	North	Center	Lisbon	Alentejo	Algarve
MEN						
50	€22,868	€21,271	€21,091	€32,376	€21,147	€19,808
51	€23,875	€21,066	€20,753	€32,081	€21,659	€18,518
52	€24,609	€21,357	€20,290	€31,991	€22,772	€19,523
53	€24,688	€21,497	€20,834	€32,895	€22,182	€18,722
54	€25,831	€21,942	€20,475	€31,422	€24,079	€19,314
55	€25,624	€22,005	€21,086	€32,877	€23,712	€20,063
56	€25,185	€22,005	€21,065	€33,006	€24,686	€18,943
57	€25,813	€22,665	€20,187	€33,248	€23,917	€20,398
58	€23,696	€22,757	€20,719	€34,349	€24,228	€18,808
59	€25,881	€23,593	€21,222	€34,765	€25,096	€20,406
60	€23,843	€21,586	€20,037	€32,585	€24,078	€19,509
61	€26,350	€22,616	€21,393	€36,334	€22,330	€19,943
62	€25,966	€24,180	€20,432	€34,934	€25,275	€18,210
63	€24,645	€23,053	€19,851	€33,056	€25,360	€17,408
64	€24,490	€24,474	€20,181	€34,764	€28,163	€17,266
Average (50–64)	€24,891	€22,404	€20,641	€33,379	€23,912	€19,123
WOMEN						
50	€16,290	€14,524	€13,384	€20,811	€13,740	€15,466
51	€15,880	€14,864	€13,451	€20,522	€13,811	€15,637
52	€16,709	€15,202	€13,291	€20,965	€14,281	€15,595
53	€16,165	€15,006	€13,067	€21,020	€14,068	€14,931
54	€17,526	€15,250	€13,158	€21,075	€13,612	€14,653
55	€16,504	€15,379	€13,459	€20,312	€13,731	€15,434
56	€15,437	€15,251	€13,343	€21,297	€14,298	€15,507
57	€15,557	€15,313	€12,612	€20,181	€14,629	€15,402
58	€15,359	€15,205	€13,018	€19,968	€13,632	€14,444
59	€16,881	€16,023	€14,525	€19,495	€13,140	€15,262
60	€15,727	€15,726	€13,192	€19,362	€13,180	€15,151
61	€16,103	€15,792	€12,819	€19,901	€13,359	€13,546
62	€15,954	€15,860	€13,088	€20,745	€13,340	€14,257
63	€15,629	€15,452	€13,850	€18,797	€13,298	€16,413
64	€15,460	€15,489	€13,070	€17,956	€13,426	€14,059
Average (50–64)	€16,079	€15,356	€13,288	€20,160	€13,703	€15,050
NATIONAL	€20,255					

*Note: National values are weighted averages according with each age, sex and region population size*

### Appendix 2

**Table 4:** Logistic regression analysis by type of early exit from paid employment

	Unemployment		Early Retirement		Early Exit From Paid Employment	
	*Univariable OR (95% CI)*	*Multivariable OR* *(95% CI)* ^*a*^	*Univariable OR* *(95% CI)*	*Multivariable OR* *(95% CI)* ^*a*^	*Univariable OR (95% CI)*	*Multivariable OR* *(95% CI)* ^*a*^
Knee Osteoarthritis	1.97* (1.25–3.10)	2.68* (1.58–4.53)	1.37 (0.90–2.08)	–	2.22* (1.49–3.29)	2.25* (1.42–3.59)
Age	0.99* (0.97–1.00)	0.98* (0.97–1.00)	1.03* (1.01–1.04)	1.03* (1.01–1.04)	1.01 (1.00–1.03)	1.01* (1.00–1.03)
Gender (Female)	1.31 (0.83–2.09)	1.06 (0.61–1.84)	0.75 (0.47–1.18)	0.64 (0.41–1.00)	1.27 (0.80–2.00)	1.23 (0.79–1.93)
Educational level (Ref: Primary or less)						
Medium	0.89 (0.51–1.56)	–	0.47* (0.26–0.84)	0.46* (0.23–0.92)	0.41* (0.22–0.74)	0.31* (0.18–0.55)
High	0.77 (0.44–1.34)	–	0.46* (0.26–0.80)	0.62 (0.36–1.06)	0.29* (0.17–0.49)	0.49* (0.27–0.89)
Marital Status (Ref: Single)						
Married/Consensual Union	0.85 (0.36–1.99)	–	4.38* (1.81–10.57)	4.21* (1.62–10.92)	3.11* (1.30–7.43)	5.28* (2.24–12.45)
Divorced	0.97 (0.36–2.57)	–	2.89 (0.97–8.63)	2.99 (0.97–9.22)	1.77 (0.64–4.87)	1.78 (0.69–4.58)
Widowed	0.41 (0.13–1.29)	–	6.14* (2.23–16.93)	6.08* (2.00–18.48)	3.93* (1.50–10.29)	4.04* (1.34–12.17)
Chronic Diseases						
Cardiovascular	0.88 (0.55–1.42)	–	0.97 (0.58–1.62)	–	1.04 (0.64–1.68)	–
Diabetes	0.87 (0.47–1.61)		2.25* (1.30–3.89)	1.92* (1.09–3.41)	2.78* (1.67–4.65)	–
Pulmonary	0.98 (0.43–2.24)	–	2.08 (0.83–5.21)	–	2.08 (0.84–5.15)	–
Allergy	0.86 (0.51–1.44)	–	0.70 (0.41–1.20)	–	0.76 (0.43–1.33)	–
Gastrointestinal	1.02 (0.63–1.66)	–	1.40 (0.89–2.23)	–	1.45 (0.91–2.31)	–
Neoplasic	1.27 (0.50–3.26)	–	1.61 (0.70–3.70)	–	1.51* (0.62–3.65)	
Mental	1.04 (0.64–1.70)	–	0.82 (0.54–1.23)	–	1.34 (0.91–1.99)	–
Neurologic	0.50 (0.15–1.59)	–	4.83* (2.19–10.66)	5.00* (1.95–12.83)	4.17* (1.83–9.50)	4.58* (1.40–15.00)
Household Income (Ref: ≤€500)						
>€500 and ≤ €1000	0.31* (0.18–0.55)	0.36* (0.20–0.64)	0.84 (0.43–1.62)	–	0.43* (0.23–0.80)	0.40* (0.23–0.69)
>€1000 and ≤ €2000	0.10* (0.04–0.24)	0.12* (0.05–0.28)	0.98 (0.45–2.12)	–	0.26* (0.12–0.54)	0.25* (0.12–0.53)
>€2000	0.15* (0.06–0.43)	0.18* (0.06–0.50)	0.79 (0.34–1.81)	–	0.22* (0.10–0.49)	0.25* (0.10–0.65)
Regional Controls		YES		YES		YES

*OR odds ratio, CI confidence interval, *p < 0.05*

*a - All multivariable models were adjusted for age, gender, geographic region (7 main regions: North, Center, Lisbon region, Alentejo, Algarve, Azores and Madeira), marital status, education level, household income, body mass index and chronic diseases. Cofactors were excluded in the stepwise method if p > 0.05 (except for age and gender)*

## Abbreviations

ACR: American College of Rheumatology
BMI: Body Mass Index
EQ-5D: European Quality of Life questionnaire
GDP: Gross Domestic Product
HAQ: Health Assessment Questionnaire
OA: Osteoarthritis
OR: Odds Ratio
PAF: Population Attributable Fractions
SF-36: 36-Item Short Form Survey
